# TOR complex 2 is needed for cell cycle progression and anchorage-independent growth of MCF7 and PC3 tumor cells

**DOI:** 10.1186/1471-2407-8-282

**Published:** 2008-10-03

**Authors:** Ville Hietakangas, Stephen M Cohen

**Affiliations:** 1 Temasek Life Sciences Laboratory, 1 Research Link, The National University of Singapore, 117604, Singapore; 2 European Molecular Biology Laboratory, Meyerhofstrasse 1, 69117 Heidelberg, Germany

## Abstract

**Background:**

AKT signaling promotes cell growth, proliferation and survival and is hyperactivated in many cancers. TOR complex 2 (TORC2) activates AKT by phosphorylating it on the 'hydrophobic motif' site. Hydrophobic motif site phosphorylation is needed only for a subset of AKT functions. Whether proliferation of tumor cells depends on TORC2 activity has not been thoroughly explored.

**Methods:**

We used RNAi-mediated knockdown of rictor to inhibit TORC2 activity in MCF7 and PC3 tumor cells to analyze the importance of TORC2 on proliferation of tumor cells.

**Results:**

TORC2 inhibition reduced proliferation and anchorage-independent growth of both cell lines. Rictor depleted cells accumulated G1 phase, and showed prominent downregulation of Cyclin D1.

**Conclusion:**

This study provides further evidence that inhibition of TORC2 activity might be a useful strategy to inhibit proliferation of tumor cells and subsequent tumor growth.

## Background

AKT signaling promotes cell growth, proliferation and survival and is hyperactivated in numerous cancers (Reviewed in [1,2]). AKT kinase activity is principally determined by the level of phosphatidylinositol-3,4,5-triphosphate (PIP3) in the plasma membrane of cells, which is generated by phosphatidylinositol-3-kinase (PI3K) upon stimulation of receptor tyrosine kinases. PI3K is counteracted by the lipid-phosphatase and tumor suppressor PTEN, which converts PIP3 back to PIP2 (Reviewed in [1,2]). When PIP3 levels are elevated, AKT is recruited to the plasma membrane and phosphorylated in the activation loop by PDK1. In addition, AKT contains a highly conserved C-terminal hydrophobic motif (HM) that must also be phosphorylated for full AKT activation in vitro [3].

Recent studies in mammals and Drosophila have demonstrated that TORC2 is responsible for HM site phosphorylation [4-6]. Surprisingly, TORC2-mediated phosphorylation only affects a subset of AKT functions. MEFs lacking essential TORC2 components show reduced phosphorylation of FOXO, but not reduced phosphorylation of TSC2 or GSK-3, although all three are well-established AKT targets [7,8]. In Drosophila, TORC2 loss-of-function phenotypes are substantially different from those of the other AKT pathway members [6]. While Drosophila AKT and its upstream regulators, such as PI3K and PDK1, are essential for viability and cell growth, flies lacking TORC2 are viable and display only minor growth impairment under standard growth conditions. However, TORC2 is required for tissue overgrowth upon hyperactivation of AKT signaling, e.g. in the case of PTEN loss-of-function. This suggests that TORC2 inhibitors might be a useful for treating cancers that depend of high AKT signaling. Since TORC2-mediated phosphorylation is essential for only a subset of AKT functions, it is possible that targeting TORC2, instead of other AKT pathway members, would minimize unwanted consequences resulting from more general inhibition of AKT activities.

In order to evaluate the potential of TORC2 inhibition in cancer treatment, it is important to analyze which AKT functions depend on TORC2 in malignant cells. Here we have analyzed the effects of TORC2 inhibition on proliferation and anchorage independent growth in two different tumor cells, MCF7 breast cancer and PC3 prostate cancer cells. Inhibition of TORC2 activity by knockdown of an essential component, Rictor, inhibited cell cycle progression, cell proliferation and anchorage-independent growth in both cell types. Our results suggest that inhibition of TORC2 activity might be a useful strategy to inhibit proliferation of tumor cells and subsequent tumor growth.

## Methods

### Cell culture and treatments

MCF7 and PC3 cells were maintained in DMEM with 10% FCS and penicillin/streptomycin in humidified 5% CO_2 _atmosphere at 37°C. The siRNAs targeting human *rictor *were Hs_AVO3_1 (target sequence: AAACAAGGCTGTGATTCTA) and Hs_AVO3_2 (target sequence: AAAGACTACAGCAACAAAGAA; Qiagen). The negative control (non-silencing) siRNA had target sequence AATTCTCCGAACGTGTCACGT. siRNAs were transfected by using HiPerFect reagent (Qiagen) according to manufacturer's protocol. For AKT kinase assays, cells were treated with Insulin (Sigma, 10 μg/ml) and wortmannin (Sigma, 50 nM) for 20 min.

### Western blotting and AKT kinase assay

After treatments cells were washed once with cold PBS and lysed by boiling in Laemmli sample buffer, resolved on SDS-PAGE, transferred to nitrocellulose membrane and blotted with the following antibodies: anti-AKT phospho-S473, anti-AKT, anti-Cyclin D1 (Cell Signaling Technology), anti-Rictor (Bethyl Laboratories), anti-GAPDH (Santa Cruz Biotechnology). AKT kinase assay was purchased from Cell Signaling Technology and used according to the manufacturer's protocol. The intensities of the phospho-GSK3 bands were quantified by using the ImageJ software (NIH #3877). The total levels of GSK-3 crosstide fusion protein were visualized by Coomassie staining.

### Proliferation and cell death assays

Cells were plated at low density, transfected with siRNAs and allowed to proliferate for 2 days. After that, cells were trypsinized, diluted, plated, re-transfected, and allowed to proliferate another four days. Cells were counted with a counting chamber. For analyzing the amount of cell death, cells were seeded on chambered slides and transfected with the siRNAs for 4 days. Cells were fixed with 4% paraformaldehyde and nuclei were stained with DAPI. Cells were imaged by confocal microscopy and condensed nuclei were calculated. Cell death and nuclear condensation in MCF7 cells was induced by staurosporin treatment (1 μM/3 h).

### Soft agar assay

0.5% agar (1.5 ml/35 mm plate) containing DMEM, 10% FCS, and penicillin/streptomycin was used as base agar. Two days after siRNA transfection, 5000 cells were seeded into 1.5 ml of 0,35% medium-containing agar that was plated on top of the base agar. The plates were incubated in humidified 5% CO_2 _atmosphere at 37°C for 21 days, stained with 0.5 ml of 0.005% Crystal Violet for 1 h and counted using a microscope.

### Cell cycle analysis

Three days after transfection cells were collected, washed by PBS and fixed with 70% ethanol on ice for 1 h. Fixed cells were stained with propidium iodine (20 μg/ml) in the presence of RNAse A (200 μg/ml) in PBS with 0.1% Triton X-100 for 15 min in 37°C. Stained cells were analyzed by flow cytometry.

## Results

### Rictor depletion inhibits AKT phosphorylation and activity in MCF7 and PC3 tumor cells

**Figure 1:**
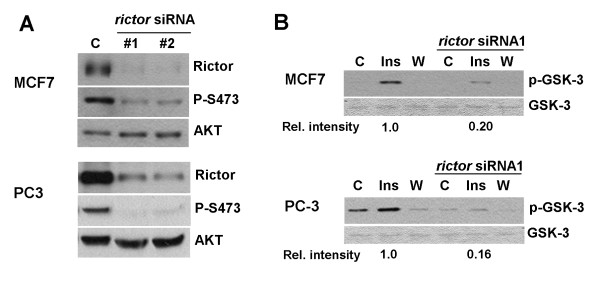
**Rictor depletion reduces AKT HM phosphorylation and inhibits AKT kinase activity**. (A) MCF7 and PC3 cells were treated for five days with two independent siRNAs against rictor or with a control siRNA not targeting any human protein. Cells were lysed by boiling in sample buffer and proteins resolved by SDS-PAGE. Levels of Rictor expression and AKT phosphorylation and expression were analyzed by immunoblotting with anti-Rictor, anti-AKT and antibody specific for the phosphorylated form of the HM site, P-S473. (B) Cells were treated for five days with rictor siRNA, treated with insulin (Ins) or wortmannin (W) or left untreated (C). Cells were lysed and AKT was immunopurified with immobilized AKT antibodies. To measure AKT kinase activity, the immunoprecipitates were incubated with paramyosin-fused GSK-3 crosstide in the presence of ATP. The level of phosphorylation was analyzed by blotting with antibodies specific to phospho-GSK-3. Total GSK-3 level is shown as a loading control.

In order to analyze the importance of TOR complex 2 for growth of tumor cells, we sought to prevent its activity by downregulating Rictor, an essential component of TORC2 [9,10]. We used two independent siRNAs directed against rictor mRNA and, as a control, an siRNA not targeting any human proteins. Both rictor siRNAs efficiently lowered Rictor protein levels in MCF7 and PC3 cells, compared to cells transfected with control siRNA (Fig. 1A).

TORC2 phosphorylates AKT on the hydrophobic motif site, which led us to analyze the effect of Rictor knockdown on this phosphorylation event. Immunoblot analysis showed that Rictor depletion led to strong inhibition of AKT hydrophobic motif phosphorylation in both cell lines, whereas total levels of AKT remained unaltered (Fig. 1A). To directly analyze the effect of Rictor depletion on AKT kinase activity, we performed a kinase assay, by immunoprecipitating AKT and assaying its ability to phosphorylate in vitro a protein containing a GSK-3 peptide harboring a consensus phosphorylation site for AKT. As expected, AKT kinase activity was induced when cells were treated with insulin and inhibited by PI3K inhibitor wortmannin (Fig. 1B). In line with the strongly reduced AKT hydrophobic motif phosphorylation, the insulin-induced AKT kinase activity was reduced to ~20% in both cell lines upon Rictor depletion (Fig. 1B).

### Inhibition of tumor cell proliferation and anchorage independent growth upon Rictor depletion

**Figure 2:**
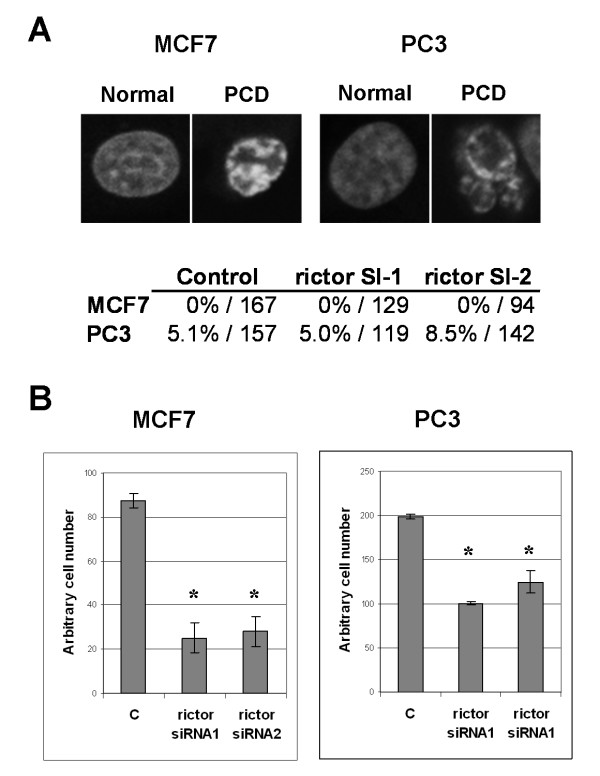
**Inhibition of TORC2 reduces proliferation of MCF7 and PC3 cells**. (A) The amount of programmed cell death (PCD) was analyzed by calculating the percentage of cells displaying nuclear condensation 4 days after siRNA treatments. In the table the percentage of condensed nuclei and the number of cells analyzed is displayed. Note that MCF7 cells lack functional caspase-3, which makes them resistant and affects their mode of cell death [20]. When treated with the commonly used cell death inducer, staurosporin, MCF7 cells undergo nuclear condensation without the nuclear fragmentation typical of normal apoptotic cells (upper panel). (B) MCF7 and PC3 cells were plated at low density and transfected with two independent rictor siRNAs or with a control siRNA. Cells were allowed to proliferate for two days until they reached near confluency, they were trypsinized, diluted and replated at low density and retransfected with the siRNAs. Cells were allowed to proliferate another four days until the control cells were nearly confluent. Cells were trypsinized, resuspended and counted with a counting chamber. All samples were done in three replicates. * Students t-test p < 0.01.

As an initial assessment for the effects of TORC2 inhibition, we analyzed the amount of cell death and the rate of proliferation of MCF7 and PC3 cells upon Rictor depletion. Controlled amounts of siRNA-transfected cells were plated at low density and allowed to proliferate for several days. During the growth period, rictor siRNAs did not cause significant increase in cell death, observed either by the extent of cell detachment (data not shown) or by analyzing nuclear morphology (Fig. 2A). Intriguingly, cell counts indicated that Rictor depletion strongly inhibited proliferation of MCF7 cells (Fig. 2B). Proliferation of PC3 cells was also significantly inhibited, albeit less strongly than in the case of MCF7 cells (Fig. 2B).

**Figure 3:**
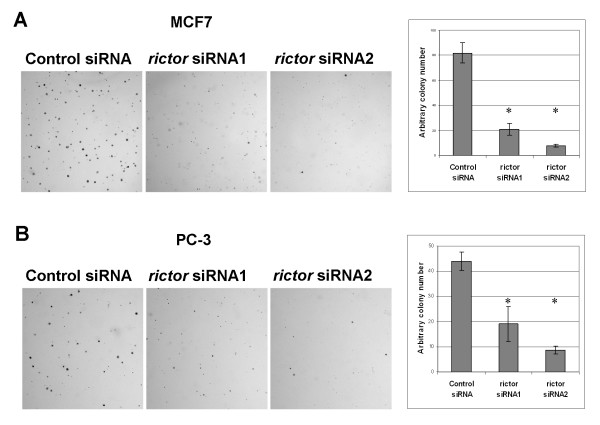
**Inhibition of anchorage independent growth upon Rictor depletion**. (A) MCF7 and PC3 cells were transfected with the siRNAs. Two days after transfection cells were detached with trypsin and seeded into soft agar. Colonies were allowed to grow for three weeks after which they were stained with crystal violet. (B) The number of visible colonies in a defined area was counted in three replicate samples. * Student's t-test p < 0.01.

Anchorage-independent growth is a hallmark of transformed cells that correlates well with their ability to be invasive and metastatic. AKT activity is crucial for tumor growth, not only because it promotes proliferation, but because it allows anchorage-independent growth by preventing detachment-induced apoptosis, anoikis [11]. Therefore, we performed a more stringent test to evaluate the importance of TORC2 in respect to tumor cell growth by analyzing the anchorage-independent growth of the MCF7 and PC3 cells after Rictor depletion. The control siRNA-treated cells formed colonies in soft agar (Fig. 3A, B), but transfection of rictor siRNA almost totally abolished the formation of colonies, demonstrating that TORC2 activity is required for anchorage-independent growth of MCF7 and PC3 cells (Fig. 3A, B).

### Accumulation of cells in G1 phase and downregulation of Cyclin D1 upon *rictor *knockdown

**Figure 4:**
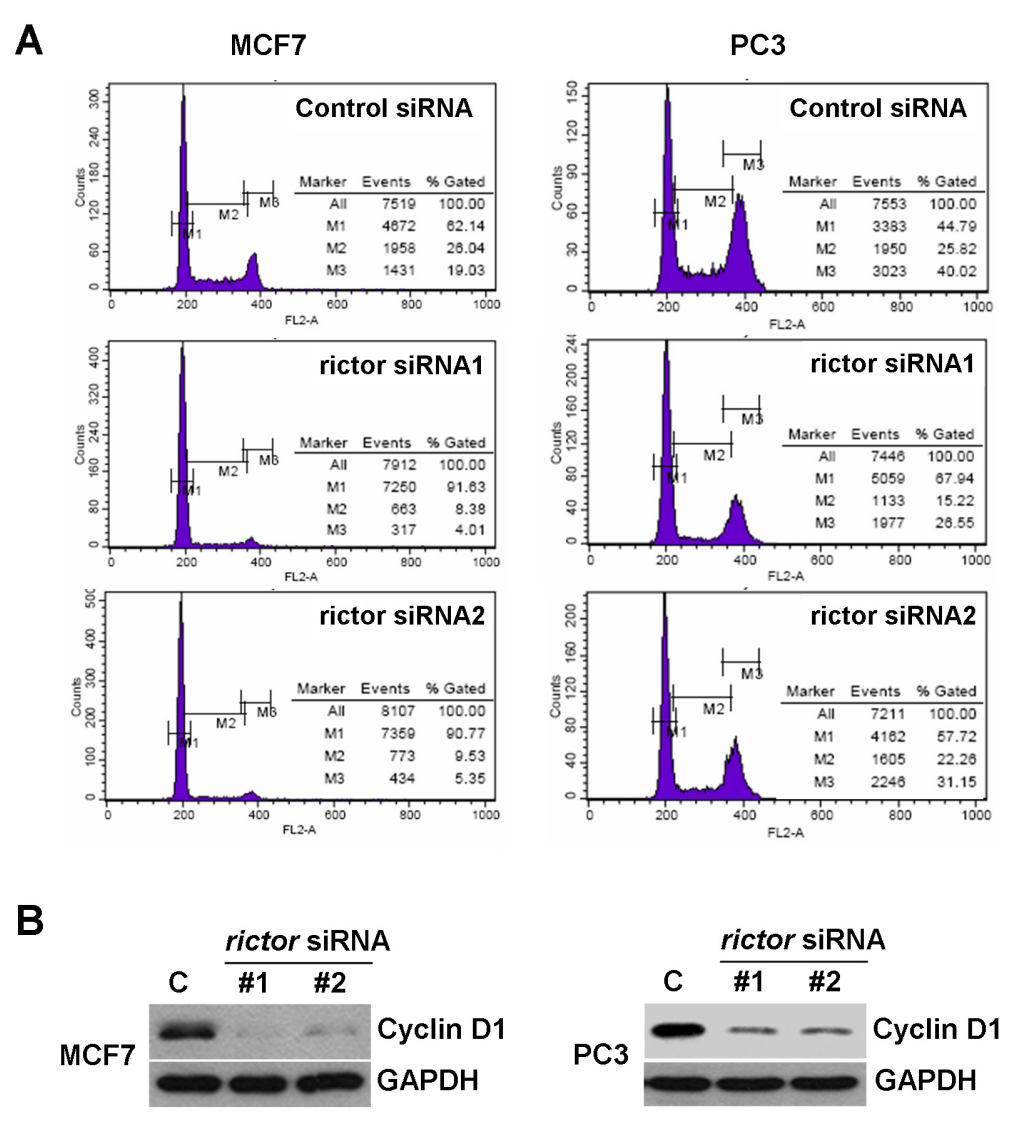
**Accumulation of cells in G1 phase upon rictor depletion correlates with downregulation of Cyclin D1**. (A) Flow cytometry histograms showing accumulation of cells in G1 phase after depletion of rictor. Cells were treated with siRNAs for five days, fixed with ethanol, stained with propidium iodine. DNA content was analyzed by flow cytometry. M1: G1, M2: S, M3: G2/M phase. (B) Cells were treated with control or rictor siRNAs for five days. Cyclin D1 levels were analyzed by immunoblotting with anti-CyclinD1. GAPDH was used as loading control.

Previous work has shown that AKT activity is needed for G1/S progression during the cell cycle, which can be explained, at least partially, by regulation of Cyclin D1 levels [12], reviewed in [13]. To examine whether the reduced rate of proliferation upon TORC2 inhibition might be explained by inhibition of cell cycle progression, we analyzed the effects of Rictor depletion on the cell cycle profile. Both MCF7 and PC3 cells displayed a clear enrichment of cells in G1 when treated with the siRNAs against Rictor (Figure 4A). MCF7 cells showed a shift from ~60% to > 90% of cells in G1. The effect was similar, but less pronounced in PC3 cells. We next analyzed Cyclin D1 levels by immunoblotting after Rictor depletion. Rictor depletion strongly reduced the expression of Cyclin D1 (Figure 4B), suggesting that phosphorylation of the hydrophobic motif of AKT is needed to maintain Cyclin D1 expression and subsequent cell cycle progression.

## Discussion and conclusion

In order to evaluate the potential of TORC2 inhibition as a strategy to impair tumor cell proliferation, we have analyzed the consequences of Rictor depletion in two distinct tumor cell lines MCF7 and PC3. Inhibition of TORC2 activity prevented proliferation and anchorage independent growth of both MCF7 and PC3 cells, although MCF7 were more sensitive to reduced TORC2 activity. Analysis of cell cycle profiles revealed a clear enrichment of cells in G1 phase in TORC2-inhibited cells, which coincided with a strong downregulation of Cyclin D1. These findings suggest that loss of HM site phosphorylation may be sufficient to mimic the effects of a more global inhibition of AKT activity in this context, although we cannot rule out the possibility of AKT-independent effects. In sum, our study shows that proliferation and anchorage-independent growth of tumor cells can be efficiently prevented by inhibiting TORC2, which therefore could be an effective strategy for treatment of cancers that depend on high AKT activity.

Which AKT functions are dependent on TORC2-mediated HM phosphorylation? Several studies provide evidence that two downstream effectors of AKT, FOXO1/3a and TORC1, respond differently to loss of AKT HM phosphorylation. In both Drosophila and mammalian systems, loss of TORC2 activity reduces phosphorylation-mediated inhibition of FOXO, whereas TORC1 activity is unaffected by loss of AKT HM phosphorylation [4,6-8]. This difference is reflected in the Drosophila TORC2 loss-of-function phenotypes. While loss of AKT, Rheb or TOR cause a clear reduction of cell size [14-16], inhibition of TORC2, and consequently AKT HM phosphorylation, does not (VH & SC, unpublished observation). FOXO1/3a, on the other hand, has been shown to be important for regulation of resistance to oxidative stress and other apoptotic stimuli, and indeed mammalian cells lacking TORC2 or AKT HM phosphorylation are hypersensitive to apoptotic stimuli induced by H_2_O_2 _[7], indole-3-carbinol [17] or etoposide [5]. The effect of TORC2 on cell proliferation has been studied in fibroblasts derived from knock-out mouse models of essential TORC2 components Rictor and Sin1 with controversial results. Shiota and coworkers analyzed proliferation of Rictor-deficient MEFs and showed that the cells displayed modestly slower proliferation compared to wild type [18], whereas Jacinto and coworkers analyzed sin1-deficient MEFs, and found that they proliferated similarly to wild type cells [7]. In the two tumor cell lines analyzed here, acute loss of TORC2 activity clearly slowed proliferation by slowing or blocking cells in G1 phase of the cell cycle. Interestingly, a recent study showed that many gliomas overexpress Rictor and have elevated TORC2 activity that contributes to their tumorigenity [19]. Results obtained in our study suggest that the potential of preventing tumor growth by inhibition of TORC2 is not only limited to gliomas, but can be used to inhibit growth of other types of tumors as well.

Sarbassov and coworkers discovered that prolonged treatments with rapamycin or its derivatives can also inhibit TOR complex 2, in addition to the well-established inhibition of TORC1 [17]. Therefore, rapamycin derivatives might be useful in preventing growth of tumors dependent on AKT HM phosphorylation. However, strong inhibition of TORC1 may have undesirable consequences. Therefore development of small-molecular inhibitors specific to TORC2 is necessary.

## Competing interests

The authors declare that they have no competing interests.

## Authors' contributions

VH planned experiments, performed experiments, analyzed data, and wrote the manuscript. SC planned experiments, analyzed data, and wrote the manuscript. Both authors have read and approved the final manuscript.

## Pre-publication history

The pre-publication history for this paper can be accessed here:


